# Associations Between Upper Extremity Activity Capacity and Strength and Post-Operative Ambulation After Geriatric Hip Fracture: A Prospective Controlled Study

**DOI:** 10.3390/jcm14041040

**Published:** 2025-02-07

**Authors:** Mahmut Tuncez, Tugrul Bulut, Yilmaz Onder, Omur Rezan Talar

**Affiliations:** Department of Orthopedics and Traumatology, Izmir Katip Celebi University Ataturk Training and Research Hospital, Izmir 35150, Turkey; drtugrulbulut@yahoo.com (T.B.); dryilmazonder@gmail.com (Y.O.); omurtalar@gmail.com (O.R.T.)

**Keywords:** hip fracture, femoral neck fracture, grip strength, cumulative ambulation score, rehabilitation, geriatric rehabilitation

## Abstract

**Background/Objectives**: This study aimed to investigate the effect of upper extremity activity capacity and hand grip strength on early post-operative ambulation in geriatric patients undergoing hip arthroplasty for hip fracture. **Methods**: This study included patients over 70 years of age who underwent cementless bipolar hemiarthroplasty for geriatric femoral neck fracture to form a homogeneous cohort. This prospective study was terminated when the number of patients reached 102 after power analysis. Demographic data, cumulative ambulation scores (CASs), quick disabilities of the arm, shoulder, and hand (QDASH) scores, and hand grip strength (HGS) were recorded both pre-operatively and post-operatively (3rd and 30th days). The presence of a linear relationship between the numerical and ordinal variables was analyzed using correlation analysis. **Results**: The mean age of the patients was 78.8 years (range: 70–93 years). There was a positive correlation between the HGS and CAS (r = 0.717, *p* < 0.05). A negative correlation was found between HGS, age (r = −0.529, *p* < 0.05), and QDASH scores (r = −0.408, *p* < 0.05). There was a negative correlation between the QDASH score, HGS, and CAS, and a positive correlation between the QDASH score and age (*p* < 0.05). **Conclusions**: This study showed a direct relationship between post-operative ambulation capacity, upper extremity activity capacity, and hand grip strength in geriatric hip fractures. While older age negatively affects this capacity, upper extremity activity capacity and hand grip strength positively affect it.

## 1. Introduction

Hip fractures are an important socioeconomic problem, with high mortality and morbidity rates in elderly patients [1,2]. The increase in life expectancy with the growing population means that this problem will continue to be an important problem both now and, in the future [2,3,4]. It is known that geriatric hip fracture patients show mental and physical decline in the post-operative period compared to the pre-operative period [5]. In particular, the decrease in physical capacity is an important factor that complicates the patient’s post-operative ambulation process and negatively affects quality of life [6,7]. As the population ages, more hip fractures occur and the socioeconomic burden increases. The delayed ambulation recovery causes economic and emotional problems for both the patient and the caregivers [8]. This is an important social problem that needs to be solved. Therefore, early independent ambulation is of vital importance [9].

Hip arthroplasty is a common treatment option for geriatric patients with hip fractures [10]. Successful post-operative ambulation/walking rehabilitation after this surgical intervention plays an important role in reducing complications and post-operative mortality rates [5,6,7,8,9,10,11]. Therefore, the early identification of patients at high risk of functional impairment/low ambulation levels after hip fracture is of great importance. Sarcopenia has been investigated in recent years to determine this, but more studies are needed to make a definitive diagnosis of sarcopenia [12,13]. However, it has been shown that HGS is a practical metric that provides information about the muscle strength of patients with hip fractures and can be easily measured with the availability of a hand dynamometer [14].

After hip replacement surgery, geriatric patients often require walking aids to regain their walking ability during the pre-fracture period, especially in the early post-operative period. The ability to use these walking aids is an important factor that affects post-operative ambulation in patients [15]. Some scoring systems have been used to evaluate the post-operative ambulation capacity of patients. However, upper extremity motor power and activity capacity are included in very few of these scoring systems [6,7,8,9,10,11,12,13,14,15,16]. However, both the ability to use walking aids and independent mobilization capacity are closely related to the functional capacity and strength of the upper extremities [16,17].

Our hypothesis was that upper extremity functional capacity and hand grip strength are important factors that enable early ambulation after geriatric hip fracture surgery.

The aim of this study was to investigate the effect of upper extremity activity capacity and hand grip strength on early post-operative ambulation in patients who underwent hip arthroplasty for geriatric hip fractures.

## 2. Materials and Methods

This study was conducted in accordance with the principles of the Declaration of Helsinki, and informed consent to participate was obtained from each participant. Approval was granted by the ethics committee of our university (IRB#140/2022), the authority that provided approval for the corresponding ethical approval code.

### 2.1. Patient Inclusion

**Figure 1:**
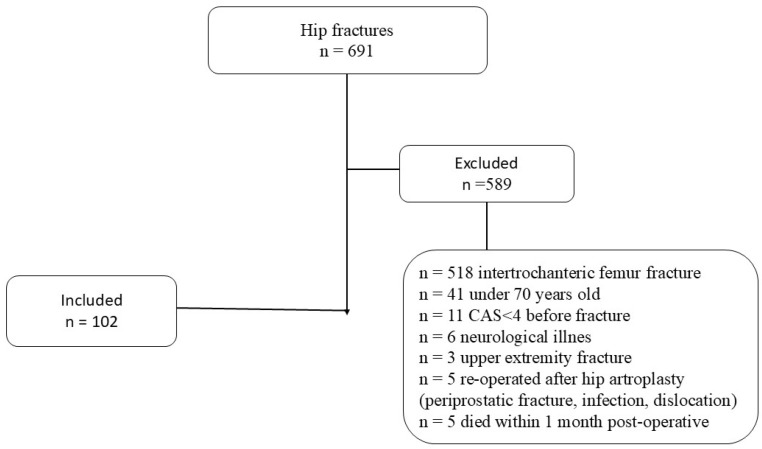
Flowchart of participants throughout this study.

We prospectively analyzed 691 patients who underwent surgery for hip fractures between September 2022 and April 2024. The exclusion criteria were the following: patients under 70 years old; hip fracture other than femoral neck fracture; concomitant fracture or additional organ injury such as thoracic, cranial, and abdominal; surgical treatment other than hemiarthroplasty; body mass index (BMI) less than 18.5 or higher than 30; reoperation due to post-operative complications; known neurologic disease; history of concomitant or previous upper extremity fracture or dislocation; history of upper extremity surgery for any reason; immobility or inability to walk independently without support in the pre-fracture period; and a cumulative ambulation score (CAS) < 4. The reason why we did not include patients with a low or high body mass index in the study was to homogenize the patients and because sarcopenia or endocrinological diseases before the fracture would affect post-operative physical activity. The reason why we excluded patients who were immobile before the fracture is that their joint range of motion and muscle mass may affect the scores we used in our study. A total of 102 patients who did not meet these criteria were included in the study (Figure 1).

### 2.2. Evaluation of Patients

Demographic data, BMI, CAS, quick disabilities of the arm, shoulder, and hand (QDASH), and hand grip strength (HGS) values were recorded. The ambulation capacity of the patients was evaluated using the CAS both pre-operatively and post-operatively (post-operative days 3 and 30). The patients’ upper extremity functional capacity and strength were evaluated only in the post-operative period to provide optimal and prognostic evaluation results [18]. This evaluation was performed using the QDASH and HGS on post-operative days 3 and 30. The reason for our pre-operative CAS evaluation was to see if they reached the same activity levels post-operatively. The reason for our post-operative 3rd day CAS and post-operative 30th day CAS evaluation was to see the possible effect of early post-operative (wound pain, adaptation, etc.) changes in the CAS results in the 1st month. All evaluations were performed by an independent orthopedic surgeon who was unaware of the present study and had 23 years of professional experience.

The CAS is a three-item scale that assesses activities that characterize the patient’s basic mobility skills: (1) getting in and out of bed (from supine position in bed to sitting on the edge of the bed, standing or transferring to sitting on a chair placed beside the bed, and returning to the supine position in bed); (2) sitting to standing from a chair with armrests (from sitting to standing to sitting); and (3) walking indoors with the use of appropriate walking aids (e.g., high walkers on wheels, walkers, rollators, or crutches) allowed in transfer and walking if necessary [7,19,20]. Clinicians were asked to objectively rate patient mobility on a three-point scale, where 0 was “not able to, despite human assistance and verbal cueing”; 1 was “able to, with human assistance and/or verbal cueing from one or more persons”; and 2 was “able to safely, without human assistance or verbal cueing” [7,19,20]. The CASs obtained on day one (1-day score) ranged from 0 to 6, where 6 indicates a completely independent ambulatory level.

With QDASH, in addition to being able to perform some daily physical and social activities, an opinion was reached about the functional capacity of the upper extremity by questioning the severity of symptoms, such as pain. With this scoring system, upper extremity function receives a total score between 0 and 100 [21]. The lower the QDASH score, the higher the upper extremity activity capacity.

The HGS evaluates the grip and grasp strength of the hand. The HGS was measured using a Jamar hand dynamometer (Asimow Engineering, Los Angeles, CA, USA). Assessment using a hand dynamometer has been shown to be a reliable and valid method among hospitalized geriatric patients, and there is no difference between assessments performed in the sitting or supine position [22,23]. Therefore, all patients were in the supine position during the measurement and were warned to apply the highest possible force so that the results were not affected by the patient position, and a standardized measurement technique could be applied. The highest value obtained after three assessments in the dominant hand was recorded in kilograms (kg) to be used for analysis.

### 2.3. Surgical Technique and Follow-Up Protocol

**Figure 2:**
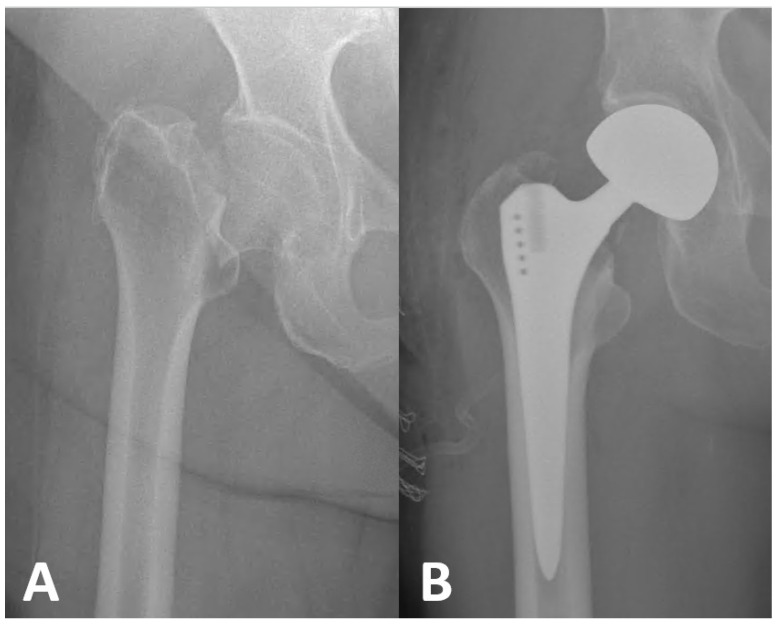
(**A**) Pre-operative anteroposterior radiograph of femoral neck fracture in a 72-year-old geriatric patient. (**B**) Post-operative anteroposterior radiographic view of this patient who underwent hip bipolar hemiarthroplasty (size 46 mm head and size 5 femoral stem) using a cementless femoral stem.

All patients underwent bipolar hip hemiarthroplasty using anterolateral Harding’s exposure under spinal anesthesia with 1 g of cefazolin prophylaxis. A cementless femoral stem was applied to all patients during the surgery (Figure 2). A pre-operative Hemovack drain was placed and removed at the post-operative 24th hour. All patients received three additional doses of cefazolin in the first 16 h post-operatively. All patients received 5000 IU of low-molecular-weight heparin subcutaneously daily for 30 days post-operatively. All patients were allowed to ambulate on the first day after surgery using a walker and bearing as much weight as they could tolerate, accompanied by a physiotherapist. Patients who had no additional problems during the post-operative follow-up were discharged. The same home-based rehabilitation program was administered to all patients after discharge. This program, which included bed mobilization and walking training, was taught to both the patients and their relatives and/or caregivers, and the maximum compliance and continuity of the patients with this program was ensured. No additional upper extremity rehabilitation program was provided to any patient to avoid affecting the results of the study.

### 2.4. Statistical Analysis

The sample size was calculated using PASS/NCSS (version 26). With a power of 90%, type 1 error level of 5%, and AUC = 0.50, the sample size was calculated as 85 people for an AUC = 0.70 value, and the study was planned to be conducted with a total of 102 patients by taking a 20% reserve sample (17 patients). Analyses were performed using the IBM SPSS Statistics for Windows, version 25.0 (IBM Corp. Released 2017, Armonk, NY, USA), package program. Numerical and ordinal variables were summarized using the median, minimum, and maximum values, while categorical variables were summarized using numbers and percentages. The conformity of numerical variables to a normal distribution was evaluated using the Kolmogorov–Smirnov test. The presence of a linear relationship between the numerical and ordinal variables was analyzed using Spearman’s rho correlation analysis, and linear regression analysis was performed for the post-operative CAS in the 1st month.

## 3. Results

Of the 102 patients included in the study, 65 (63.7%) were female and 37 (36.3%) were male. The mean age was 78.83 years (range: 70 to 93), and the mean BMI was 25.48 (range: 19 to 29). The average interval from admission to surgery was 3.80 days (range: 1–5 days), and the total hospital stay was 7.97 days (range: 3–13 days). The mean CAS was 5.45 (range: 4–6) pre-operatively, 3.24 (range: 0–6) on the third post-operative day, and 4.01 (range: 1–6) on the thirtieth post-operative day. The mean QDASH score was 35.18 (range: 0–84) on the third post-operative day and 36.15 (range: 0–96) on the thirtieth post-operative day. The mean HGS was 16.79 kg (range: 2.2–45 kg) on the third post-operative day and 17.20 kg (range: 3–46 kg) on the thirtieth post-operative day (Table 1).

According to the correlation analysis, there was a positive correlation between the HGS and CAS and a negative correlation between the HGS, age, and QDASH score (*p* < 0.05). It was shown that all CAS values of patients with high HGS were statistically significantly higher than those with low HGS. Age was negatively correlated with HGS and CAS and positively correlated with QDASH. There was a negative correlation between the QDASH score, HGS, and CAS, while there was a positive correlation between the QDASH score and age (*p* < 0.05). There was no correlation between the body mass index, time until surgery, time after surgery, total hospital stay, CAS, HGS, and QDASH scores (*p* > 0.05) (Table 2).

According to the multivariate regression analysis results for the post-operative 1st month CAS, the post-operative QDASH and HGS were found to be statistically significant (Table 3).

## 4. Discussion

This study was performed on patients over 70 years of age who underwent cementless bipolar hemiarthroplasty for geriatric femoral neck fractures to create a homogeneous cohort. The results of this study show a direct relationship between post-operative ambulation capacity, upper extremity activity capacity, and hand grip strength in geriatric hip fracture patients. Advanced age negatively affects this capacity, whereas upper extremity activity capacity and hand grip strength positively affect it. These data imply that HGS and QDASH can be used as predictive factors for evaluating the post-operative ambulation capacity of geriatric patients with hip fractures. The higher the HGS value and the lower the QDASH value of a geriatric hip fracture patient, the higher the post-operative ambulation capacity of the patient. On the other hand, a surgeon who has a geriatric patient with the opposite scores should pay more attention to the patient’s post-operative exercises; following the patient more closely and applying the rehabilitation program persistently and diligently would be a much wiser treatment management. This means that the patient’s post-operative ambulation capacity will be low.

Older age is directly related to the post-operative mental and physical decline observed in geriatric patients [24,25,26]. This explains the decline in ambulation capacity observed after fragility fracture. In this study, we found a negative correlation between age and post-operative ambulation capacity, consistent with the literature [5,11,15,25]. Although we found that age lost statistical significance according to the multivariate analysis result, we still believe that age is an important factor. We attribute the fact that age lost significance as a result of this analysis to the fact that this study was conducted only on geriatric patients over the age of 70. In the early post-operative mobilization of geriatric patients, walking with support is the basic rehabilitation approach [27,28]. Therefore, we believe that upper extremity rehabilitation and strengthening programs should be included in post-operative follow-up protocols for geriatric patients to be able to ambulate independently after treatment. Although older age is one of the main obstacles for clinicians to achieve rehabilitation and strengthening goals, surgeons should be careful and insistent on this issue. The importance of upper extremity function and strength is obvious in the balanced and correct use of walking aids that support ambulation.

The early identification of patients at high risk for functional impairment after hip fracture is of great importance for acute care planning, rehabilitation strategies, and hospital evaluation and management after discharge [28,29]. The surgeon needs objective and concrete data to identify these patients early. In geriatric patients with hip fractures, time works against the patient. Therefore, surgeons must make fast and accurate decisions within a limited timeframe to determine and implement appropriate treatment strategies. For this, it is very important to know and evaluate the predictive factors that can be used to predict early ambulation capacity and to use these factors widely in daily practice. They will be a guide for surgeons to identify high-risk patients and to start preventive treatment protocols as soon as possible. When the relevant literature is examined, it is accepted that many factors affect the post-operative functional results of geriatric patients, although there is no full consensus [28,29,30,31]. In this study, we found that worse QDASH and HGS scores of geriatric hip fracture patients result in worse CASs in the early post-operative period. We interpreted this result as QDASH and HGS being usable as predictive factors for determining the post-operative ambulation capacity of geriatric patients with hip fractures.

The CAS is a tool that facilitates interdisciplinary communication and has proven its validity in the evaluation of patients with hip fractures, including those with cognitive dysfunctions [7,19]. The CAS helps in the early prediction of short- and long-term mortality, as well as in the rehabilitation process [7,19]. In our study, the pre-operative and post-operative activity levels of the patients were evaluated using the CAS. There was a regression in the post-operative CASs compared to the pre-operative CASs. We believe that one of the most important factors in this study was older age. This is because there was a negative correlation between the CAS and older age in the present study. We also found that geriatric patients with high CASs before fracture had a higher physical capacity in the first post-operative month. There was a positive correlation between this capacity and HGS. HGS is a parameter that indicates upper extremity strength. Especially in geriatric patients, HGS is accepted as an objective indicator of general body strength and resistance, including lower extremity muscle strength [32,33,34]. Significant correlations were demonstrated between HGS and recovery in elderly patients undergoing surgery after hip fracture [34,35]. Therefore, we believe that HGS is one of the most important predictive factors for post-operative ambulation capacity in geriatric patients with hip fracture. Therefore, we suggest that HGS should be used in the daily routine as a predictive factor in the treatment management of geriatric patients with hip fractures. HGS is an important, objective, and concrete indicator for surgeons undertaking the treatment process and can indicate sarcopenia, and especially the patient’s post-operative ambulation capacity. Increasing the awareness of surgeons on this issue may allow for the early diagnosis of such patients and the necessary measures to be taken in advance. This is an important factor that medically protects the surgeon. In addition, adding programs that increase upper extremity activity capacity and strength to rehabilitation programs after hip fractures may contribute to the correct management of this process. This is very important for post-operative independent mobilization of geriatric hip fracture patients.

The most important limitations of this study are the relatively small number of patients and the fact that it was conducted in a single center. Another limitation of this study is that the results were not compared with those of a group of patients who underwent a rehabilitation program to increase upper extremity functional capacity and strength. In the future, testing the effects of upper extremity rehabilitation and strengthening programs with prospective randomized controlled trials may provide more objective data. Nevertheless, the fact that this study was conducted on a standardized homogeneous cohort and had a prospective design is a strength that makes this study important.

## 5. Conclusions

In conclusion, upper extremity activity capacity and hand grip strength are important factors that affect early post-operative ambulation in geriatric patients after hip fracture surgery. The scoring systems showing this capacity and strength are directly related to the patient’s post-operative ambulation capacity, and the surgeon can accept and use these scores as predictive factors. Furthermore, the early identification of such patients and the incorporation of programs that increase upper extremity activity capacity and strength into the post-operative rehabilitation process may contribute to the post-operative ambulation capacity of these patients. This is important for improving the quality of life of geriatric patients after hip fracture surgery.

## Figures and Tables

**Table 1:** Summary statistics for patients’ demographic and clinical variables.

	x ± sd	M (Min–Max)
Age (year)	78.83 ± 6.99	78.50 (70–93)
BMI	25.48 ± 3.01	25.35 (19–29)
Female	65	-
Male	37	-
Time until surgery (day)	3.80 ± 1.06	4 (1–5)
Time after surgery (day)	4.17 ± 2.06	4 (1–8)
Total hospital stay (day)	7.97 ± 2.36	8 (3–13)
Preop CAS	5.45 ± 0.74	6 (4–6)
Postop CAS (3rd day)	3.24 ± 1.02	3 (0–6)
Postop CAS (1st month)	4.01 ± 1.05	4 (1–6)
Postop QDASH (3rd day)	35.18 ± 33.02	35.10 (0–84.09)
Postop QDASH (1st month)	36.15 ± 33.90	38.25 (0–96)
Postop HGS (kg) (3rd day)	16.79 ± 9.99	15 (2.2–45)
Postop HGS (kg) (1st month)	17.20 ± 10.20	15.2 (3–46)

x: mean; sd: standard deviation; M: median; min: minimum; max: maximum. CAS: cumulated ambulation score; QDASH: quick disabilities of the arm, shoulder, and hand; HGS: hand grip strength. BMI: body mass index; Preop: pre-operative; Postop: post-operative; kg: kilogram.

**Table 2:** Correlation between CAS, QDASH, HGS and other variables.

	Preop CAS	Postop CAS 3rd Day	Postop CAS 1st Month	Postop QDASH 3rd Day	Postop QDASH 1st Month	Postop HGS 3rd Day	Postop HGS 1st Month
**Age**	−0.489 ** **0.001**	−0.373 ** **0.001**	−0.417 ** **0.001**	0.530 ** **0.001**	0.560 ** **0.001**	−0.519 ** **0.001**	−0.529 ** **0.001**
**BMI**	0.003 0.979	0.156 0.118	0.061 0.544	0.087 0.386	0.097 0.396	0.040 0.689	0.060 0.689
**Time until surgery**	−0.082 0.412	−0.013 0.898	−0.134 0.178	0.052 0.601	0.062 0.621	−0.061 0.540	−0.081 0.540
**Time after surgery**	0.002 0.988	0.111 0.267	0.082 0.413	0.088 0.377	0.098 0.377	0.126 0.208	0.156 0.208
**Total hospital stay**	−0.010 0.921	0.059 0.556	0.012 0.907	0.065 0.518	0.075 0.538	0.090 0.367	0.190 0.367
**Preop** **CAS**	x	0.450 ** 0.001 *	0.485 ** **0.001**	−0.532 * **0.001**	−0.532 * **0.001**	0.483 ** **0.001**	0.513 ** **0.001**
**Postop CAS** **3rd day**	0.450 ** 0.001 *	x	0.699 ** **0.001**	−0.504 ** **0.001**	−0.494 ** **0.001**	0.405 ** **0.001**	0.415 ** **0.001**
**Postop CAS** **1st month**	0.485 ** **0.001**	0.699 ** **0.001**	x	−0.589 0.001	−0.579 ** **0.001**	0.617 ** **0.001**	0.717 ** **0.001**
**Postop QDASH** **3rd day**	−0.552 * **0.001**	−0.504 ** **0.001**	−0.589 0.001	x	0.072 0.413	−0.418 ** **0.001**	−0.446 ** **0.001**
**Postop QDASH** **1st month**	−0.532 * **0.001**	−0.494 ** **0.001**	−0.579 0.001	0.072 0.413	x	−0.518 ** **0.001**	−0.408 ** **0.001**
**Postop HGS** **3rd day**	0.483 ** **0.001**	0.405 ** **0.001**	0.617 ** **0.001**	−0.418 ** **0.001**	−0.518 ** **0.001**	x	0.087 0.340
**Postop HGS** **1st month**	0.513 ** **0.001**	0.415 ** **0.001**	0.717 ** **0.001**	−0.446 ** **0.001**	−0.408 ** **0.001**	0.087 0.340	x

x: conflicting data. * r: correlation coefficient is significant at *p* < 0.005; ** r: correlation coefficient is significant at *p* < 0.001. Statistically significant data are highlighted in bold. CAS: cumulated ambulation score; QDASH: quick disabilities of the arm, shoulder, and hand; HGS: hand grip strength. BMI: body mass index; Preop: pre-operative; Postop: post-operative.

**Table 3:** Regression analysis for post-operative CAS in 1st month.

Model	Unstandardized Coefficients		Standardized Coefficients	t	Sig.	VIF
	B	Std. Error	Beta			
Constant	3.55	1.10	**–**	3.209	**0.002**	–
Postop QDASH 1st month	−0.01	0.00	**−0.365**	−4.018	**<0.001**	1.606
Postop HGS 1st month	0.05	0.01	**0.437**	4.857	**<0.001**	1.575
Age	0.00	0.01	0.000	−0.005	0.996	1.528

Adj. R^2^ = 0.482; Statistically significant data are highlighted in bold. CAS: cumulated ambulation score; QDASH: quick disabilities of the arm, shoulder, and hand; HGS: hand grip strength; Postop: post-operative.

## Data Availability

The data are available from the corresponding author if required.
